# Exploring opportunities with activity-based work in Swedish public workplaces—a qualitative analysis based on the socio-ecological model

**DOI:** 10.1080/17482631.2026.2728848

**Published:** 2026-09-08

**Authors:** Josefine Hansson, Erika Wall, Pär Löfstrand, Stig Vinberg

**Affiliations:** a Department of Health Sciences, Faculty of Human Sciences, Mid Sweden University, Östersund, Sweden; b Department of Psychology and Social Work, Faculty of Human Sciences, Mid Sweden University, Östersund, Sweden

**Keywords:** Socio-ecological model, well-being, activity-based work, SEM, salutogenic

## Abstract

**Introduction:**

Activity-based work (ABW) is a workplace concept that offers different work environments, letting employees choose settings that best support their specific tasks. Although research on ABW is growing, there is limited knowledge about how employees experience opportunities in these environments, particularly from a salutogenic health-promoting perspective.

**Methods:**

A qualitative design was applied using a survey with open-ended questions, completed by 448 employees in administrative roles in one public sector organization in Sweden. A combined deductive and inductive content analysis was conducted. The socio-ecological model was used as a deductive categorization matrix to organize the data into three levels, while subcategories were developed inductively within each level based on recurring patterns in the material.

**Results:**

The analysis identified six subcategories within the three levels of the socio-ecological model: intrapersonal (autonomy, well-being), interpersonal (social support, collaboration), and organizational (adapted facilities, an attractive work environment).

**Conclusions:**

The findings indicate that employees' perceived opportunities in ABW can be understood across intrapersonal, interpersonal, and organizational levels, and that these levels are closely connected. The study suggests that the socio-ecological model can be useful for understanding employee experiences in organizational settings and highlights the importance of considering factors at different levels when designing ABW environments.

## Introduction

In many countries, public sector workplaces account for the largest share of the workforce. In Sweden, around 1.5 million individuals are employed in the public sector, of a workforce numbering approximately 5 million (Swedish Association of Local Authorities & Regions (SALAR), 2024). Within the Nordic countries, the adoption of new public management strategies and the reduction of available resources have been identified as drivers of the heavier workloads and diminished job control reported in public sector workplaces (Rasmussen et al., 2025). In Swedish public sector workplaces, the psychosocial working conditions are often challenging, leading to significant health issues and a rise in employee sick leave (Aronsson et al., 2019). Studying the public sector is of interest because it employs a large proportion of the workforce in Nordic countries. Furthermore, it differs from the private sector in organizational conditions and is comparatively under-researched (Knies et al., 2024). Unlike private companies, public sector organizations are non-profit and funded by taxes, which can influence work practices and organizational conditions (Boxall et al., 2007). This study focuses on Swedish public sector workplaces, where hybrid work is common, but the findings may also be relevant for other countries with similar office practices, hybrid work arrangements, and public sector structures.

The proportion of individuals who partially or fully work from home varies significantly across European countries, ranging from approximately 20% to 80%. With around 60% of its workforce participating in remote work (Eurofound, 2025), Sweden ranks among the countries with the greatest prevalence of people working from home, which has encouraged the adoption of various alternative office solutions. For instance, in recent years, many workplaces in Sweden have adopted activity-based workplaces (ABW) (Hansson et al., 2025), often due to cost savings and to use office spaces more efficiently (Bergsten et al., 2021). In ABW, employees are expected to move between different work areas depending on the task they will perform, and no fixed workstations are assigned to them (Appel-Meulenbroek et al., 2011; Engelen et al., 2019). If the work task requires concentration, a quiet area may be chosen. On the other hand, if the task requires proximity to colleagues, an open area for communication and collaboration may be chosen (Appel-Meulenbroek et al., 2011).

The research on ABW has grown in recent years, but remains characterized by inconsistent findings and methodological inconsistencies. Systematic reviews have also reported mixed findings in regard to ABW. Engelen et al. (2019) found both positive and negative effects on health, work performance, and workplace perceptions, and Masoudinejad and Veitch (Masoudinejad & Veitch, 2023) highlighted mixed effects on organizational productivity-related factors. The findings from these reviews show the complexity of ABW and the need for research exploring different dimensions of employees' experiences. For instance, some studies highlight positive outcomes compared to open-plan offices, such as increased satisfaction with auditory privacy and aesthetics (Rolfö et al., 2018), reduced self-reported sedentary behaviour and low back pain (Foley et al., 2016), and increased collaboration across teams (Hansson et al., 2025). However, others have reported challenges, including a lack of communication within teams, unclear rules, and overcrowding (Babapour Chafi & Rolfö, 2019). In addition, much of the existing research tends to focus on productivity-related outcomes, such as perceived productivity (De Been & Beijer, 2014; Kim et al., 2016) and performance (Jahncke & Hallman, 2020; Meijer et al., 2009; Seddigh et al., 2015). This aligns with a predominantly pathogenic focus in workplace research, where mitigating risks and negative outcomes often takes precedence over exploring salutogenic opportunities to promote health and well-being (Forooraghi, 2022). The salutogenic approach concentrates on factors that contribute to a positive work experience rather than focusing on risks and challenges (Eriksson et al., 2022; Mittelmark et al., 2022). Instead of studying individuals separately from the physical and social context, the salutogenic approach examines how opportunities and resources are created and utilized within people and their everyday life context (Lindström & Eriksson, 2006). Studies on ABW with a salutogenic focus are rare (Hansson et al., 2025; Wijk et al., 2020). A Swedish study explored how employees' sense of coherence (SOC) influenced their health, well-being, and work satisfaction before and after relocating to a workplace with ABW in Sweden (Wijk et al., 2020). The results showed that if the employee had a stronger SOC, especially a sense of meaningfulness, they showed less decline in work satisfaction and adapted better to the new way of working. It was concluded that to mitigate a possible decline in satisfaction with the physical and psychosocial work environment in organizations implementing ABW, it is important to promote perceived meaningfulness in the process.

A holistic framework that can be placed under the salutogenic umbrella (Lindström & Eriksson, 2010) is the socio-ecological model (SEM) (McLeroy et al., 1988). The SEM conceptualizes the linkages between individual behaviour and social and environmental determinants. It also recognizes that human behaviour is not only the product of biological factors but is influenced by various personal and social and physical environmental factors and the interplay between them (McLeroy et al., 1988). As described by the SEM according to McLeroy, an individual's behaviour is influenced by factors on the intrapersonal (e.g., knowledge, beliefs, attitudes and skills), interpersonal (e.g., social norms, peers and family), organizational (e.g., environment and ethos), the community (cultural values and norms) and public policy (policies and laws at the local, regional or national level) (McLeroy et al., 1988; Sallis et al., 2008). These factors are influenced by the individual's physical and social environments (Salihu et al., 2015). The initial model acknowledged the many contributors to human development, but the model can also be used to focus on specific settings and contexts (van Kasteren et al., 2020). For instance, the SEM has been used in multilevel approaches to areas such as public health promotion, violence prevention, healthy college campuses, geriatric preventive health, and teachers' perceptions on physical activity among kindergarten children, to name a few (Kilanowski, 2017; Liang et al., 2024).

Despite the model being widely accepted, systematic reviews show that studies using the model seldom incorporate multilevel strategies. Most studies focus on only one or two socio-ecological levels, and there is a need to incorporate additional levels in research and interventions (Golden & Earp, 2012).

To our knowledge, no studies have been conducted regarding SEM and ABW. Previous research on ABW has primarily focused on organizational implementation (Bergsten et al., 2022), design characteristics (Forooraghi et al., 2025), and organizational productivity-related outcomes (Masoudinejad & Veitch, 2023). Recent research has shown that favourable perceptions of ABW are associated with improved employee well-being and self-reported work ability in hybrid work, and that the office environment is important even when psychosocial factors are considered (Tulenheimo-Eklund et al., 2025).

However, the model has been used in other work-related studies, such as discussing recommendations for preventing burnout from a socio-ecological perspective (Habeger et al., 2022) and using the SEM to understand workplace violence in China's health sector (Wu et al., 2022). Furthermore, a study reviewing socio-cultural and physical factors impacting physical activity among office-based workers concluded that socio-ecological models are important for understanding physical activity in office settings due to the sedentary nature of office work. The authors emphasized that interventions should not rely solely on individual motivation for behaviour change but also incorporate changes to the broader social, ecological, and physical context to create opportunities for more sustainable change (van Kasteren et al., 2020).

Since ABW is a complex and multifaceted way of organizing work environments, the SEM provides a particularly useful perspective. Previous research has often examined different aspects of ABW separately, such as physical design, implementation processes, or productivity outcomes. However, less attention has been paid to how these dimensions interact and how they are experienced by employees in their everyday work. Hence, there is a need to explore ABW from a multilevel perspective that considers how organizational, interpersonal, and individual factors interact to shape employees' experiences.

In this study, SEM is applied to understand employees' perceived opportunities in ABW at the intrapersonal, interpersonal, and organizational levels. The study builds on a salutogenic approach, which emphasizes factors that contribute to positive work experiences rather than risks or constraints. This perspective complements SEM by directing attention to the opportunities employees perceive in their work. Together, these theoretical perspectives form the foundation for the study's research questions.

## Aim and research questions

The aim of the study was to explore employees' perceived opportunities with ABW using the socio-ecological model as a framework. Three research questions will be addressed:

- What opportunities are experienced by employees at the intrapersonal level?
- What opportunities are experienced by employees at the interpersonal level?
- What opportunities are experienced by employees at the organizational level?

## Method

### Research setting

During the autumn of 2023, 620 administrative workers in a northern Swedish mid-sized municipality with approximately 60,000 inhabitants moved from traditional cell offices to a newly built office building with an ABW design. The purpose of the move was to set up the municipality's activity-based way of working, continue to gain support for this approach among employees, and develop a foundation to prepare the functionality of the building for an activity-based working method. After the relocation, the workforce was spread over three floors containing open-plan offices, three zones (creative, middle and silent zones), conference rooms, a canteen and coffee rooms. The relocation process started in 2019 with a project including workshops with employees and managers, field trips by managers to other organizations that had implemented ABW and training by working in open-plan offices. The participants had a variety of administrative roles and represented a range of municipal departments, including the Department of Health and Social Care, the Municipal Executive Office, the Social and Labour Market Department, the Department of Education and Childcare, the Department of Community Development, the Technical Department, and the Department of Culture and Leisure.

### Data collection

Employees from different administrative departments (social care, school administration, health care administration, municipality board) who had recently started working in ABW were recruited via email between February and April 2024 to participate in a survey to evaluate the new way of working. At the beginning of the survey, participants were asked to write down their thoughts on the opportunities, challenges, and risks associated with the activity-based work approach. The survey included three open-ended questions:

1. “What opportunities do you see with your workplace?”
2. “What challenges do you see with your workplace?”
3. “What risks do you see with your workplace?”

The remainder of the survey consisted of closed-ended questions regarding employees' evaluations of the social and organizational work environment. For the purpose of this study, the analysis focused specifically on responses to the first question, as it addresses the research aim of exploring perceived opportunities. The survey also included separate questions addressing challenges and risks associated with ABW. However, due to the salutogenic perspective and the aim of the study, only responses concerning perceived opportunities were included in the analysis.

In total, 450 of the 620 administrative workers responded to the open-ended question (response rate: 73%). After data cleaning (e.g., removal of responses such as “N/A” or “nothing”), 448 responses were deemed suitable for analysis.

### Participants

Among the participants, 361 were women, and 85 were men, while two participants either selected “other” or did not report their gender. The mean age of the participants was 48 years. Seventy participants reported holding a managerial position. Regarding educational attainment, 322 participants reported having completed at least three years of higher education at the university level, whereas 126 had completed upper secondary education. Among the participants, 239 answered that they had children living at home.

### Study design

The research was designed as a qualitative content analysis. A combined deductive and inductive content analysis approach was applied, as described by Elo and Kyngäs (2008). The socio-ecological model (McLeroy et al., 1988) was used as a categorization matrix to guide the initial organization of the data into predefined SEM levels. Within each level, subcategories were developed inductively based on recurring patterns in the material. This approach is suitable when prior knowledge about a phenomenon exists, and a theoretical framework is available.

### Data analysis

The analysis process began with all 448 free-text responses to the open-ended question regarding opportunities associated with ABW “What opportunities do you see with your workplace?” An initial reading was conducted to gain an overall understanding of the material and identify recurring patterns and experiences. The text material was divided into meaning units, typically sentences or short passages that were relevant for the study (Table I). Many responses were brief, often single sentences, and could be coded directly. During the initial step, a categorization matrix based on the SEM was used to sort the data using NVivo (version 1.4.1). The three main categories: intrapersonal level, interpersonal level, and organizational level were set up as coding nodes, and each meaning unit was coded according to the category it fit. In the following step, the data were reviewed for content and meaning units (excerpts from responses) were coded deductively in line with the main categories.

**Table I.** Coding framework based on the SEM.

Category	Sub-category	Code	Meaning unit
Intrapersonal level	*Autonomy*	Autonomy and flexibility	“Flexibility regarding where I want to sit and with whom”
			“Flexible in terms of working at work orat home.”
	*Health and Well-being*	Mental and physical well-being	“More movement by changing zones and moving around the house more. Better ergonomics”
			“A place that gives me energy when Ineed it.”
Interpersonal level	*Collaboration*	Collaboration with others	“Opportunity to network and collaborate on joint concerns”.
			“It becomes easier to work cross-sectorally when you meet each other on a daily basis.”
	*Social support*	Relations and interactions	“Greater social opportunities that can create enhanced well-being and collegiality than before.”
			“It's fantastic to be in a big context. The interaction between colleagues in other departments makes it incredibly easy.”
Organizational level	*Adapted facilities*	Facilities adapted for different tasks	“Lots of possibilities. Good workplaces, both secluded in quiet rooms and all other areas. Many different good surfaces to work on. Many meeting rooms of different sizes.”
			“It is fantastically nice premises with many different functions that support my way of working much better than in the old environment with cellular offices. There are much better fully equipped meeting rooms with screens, sound, and power in the tables for charging computers. The breakout area/conference creates great opportunities to have citizen dialogues, conferences. The focus rooms work great for having a conversation or Teams meeting.”
	*Attractive work environment*	Good environment to work in	“Endless possibilities. It's open, it's bright, it's nice. It's a good environment.”
			“Healthy, modernly adapted premises”

Meaning units that involved personal characteristics (e.g., beliefs, attitudes, or skills) were coded into the intrapersonal level. Responses that discussed social support and peers were coded into the interpersonal level. When the responses involved the organizational environment, it was coded into the organizational level. The coded meaning units within each category were grouped into subcategories based on similarities and differences in meaning. Several preliminary patterns and codes were identified during the analysis. The codes were compared and refined before being grouped into the subcategories that best represented the material. No additional themes were identified that were sufficiently distinct or prevalent to warrant inclusion as separate subcategories.

The first author conducted the initial coding of the material. Coding decisions, category development, and interpretations were discussed with the co-authors throughout the analysis process. Any differences in interpretation were resolved through discussion until consensus was reached. The inductive development of subcategories was conducted separately within each main category. The final sub-categories were autonomy, health and well-being, social support, collaboration, adapted facilities and an attractive work environment (Table I).

## Ethics

The study was approved by the Swedish Ethical Review Authority (registration number: 2023-06718-01), as required by Swedish national legislation (Ethical Review Act, 2003:460). Prior to completing the survey, participants were required to provide informed consent by selecting the option labelled “*I give my consent to participate in the study.”* They were further informed that their participation was voluntary and that they retained the right to withdraw from the study at any stage, including after completing the survey. The data was anonymized and stored properly according to the Swedish Act on Ethical Review of Research Involving Humans (SFS 2003:460).

## Results

**Figure 1.**
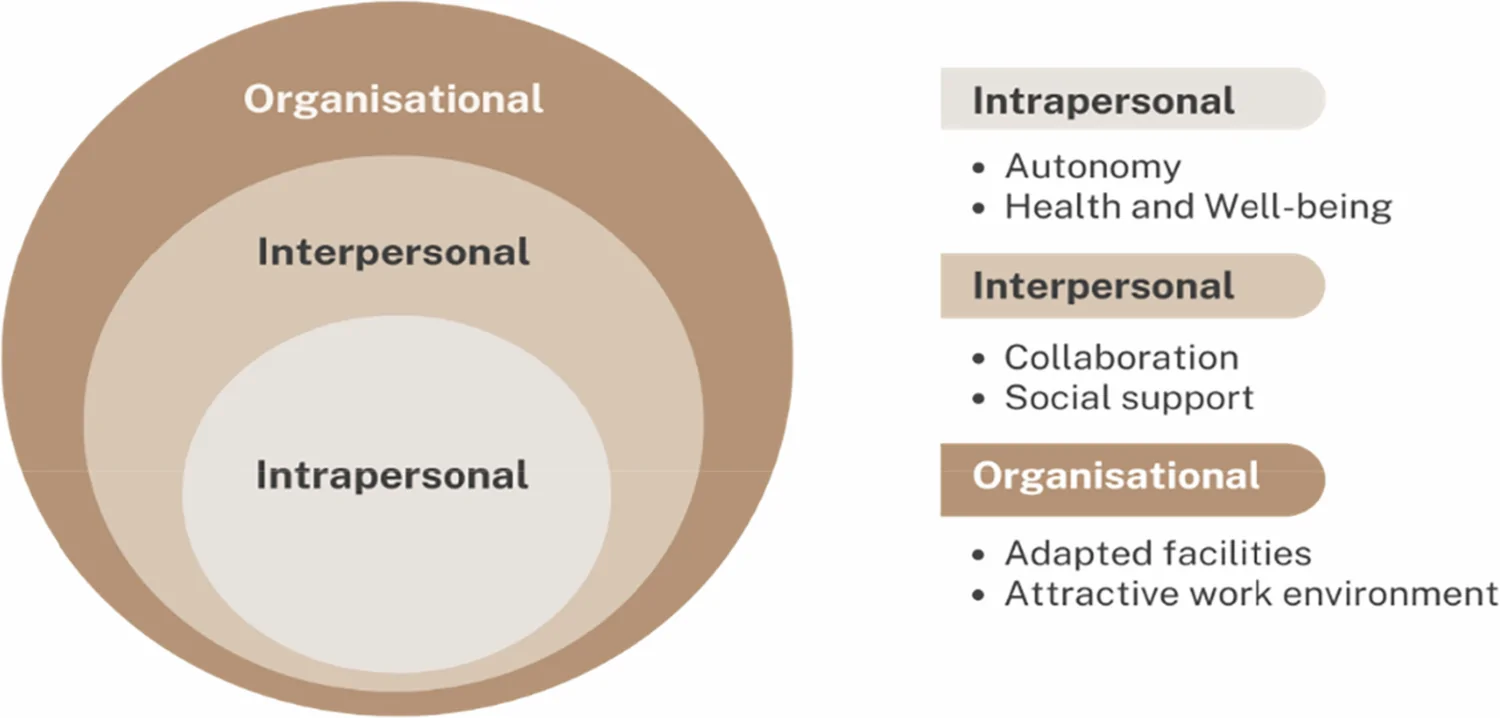
Levels within the socio-ecological model.

Within the three main categories “intrapersonal level, interpersonal level and organizational level”, six sub-categories were identified in the data. These were autonomy, health and well-being, social support, collaboration, adapted facilities, and an attractive work environment (Figure 1).

### Intrapersonal level

The first category, the intrapersonal level, focuses on individual factors such as knowledge, attitudes, perceived control, and health (McLeroy et al., 1988). This category is built on the subcategories of *autonomy* and *health and well-being*, which are connected to this level as they reflect how individuals experience ABW in terms of their health, sense of well-being, and perceived control over their work environment.

#### Autonomy

The analysis showed that the participants found that a key advantage of ABW was the ability to choose a seating area based on the work task. This was seen as important for their productivity and ability to focus. When selecting a quiet area or focus room, employees could work undisturbed and concentrate on their tasks. One participant expressed this as follows:

*"I see the opportunity to work in peace without being interrupted by colleagues. When you have a fixed place, you can almost be "kidnapped" because everyone knows where to find you if you want to discuss something, while here I can sit in quiet places or focus rooms if I want to work undisturbed."*

The flexibility was also perceived as stimulating, as it allowed employees to adapt the work environment to their own physical and psychological capabilities, and they were able to choose with whom to sit next to. The material also showed that the flexibility of working from home was seen as an advantage within the ABW concept.

*“Great flexibility. Good to be able to alternate with working from home.”*

#### Health and well-being

The material indicated that the respondents viewed increased physical activity as a potential benefit of ABW as they moved a lot when changing seating areas. It was also regarded as an opportunity for improved health, as it may help prevent back and neck issues. The following excerpt by one of the respondents illustrates this:

*"I see more opportunities for movement, which in turn may contribute to better health, for example, by reducing back and neck problems."*

The material also indicated that ABW encouraged movement, such as going outdoors for a walk in nature or walking to and from home visits. Furthermore, the opportunity to take breaks in designated rest areas was regarded as important for both physical and psychological well-being. One respondent formulated this as follows:

*"Great opportunities for breaks: going out into the forest and taking walks to and from home visits. The rest areas provide an opportunity for relaxation and calm."*

### Interpersonal level

The second category, the interpersonal level, focuses on social interactions and relationships that influence individual experiences, such as social support and collaboration (McLeroy et al., 1988). This category is built on the subcategories of *collaboration* and *social support,* which reflect how ABW facilitates interactions between colleagues, encourages knowledge sharing, and fosters a sense of community in the workplace. In the analysis, collaboration included opportunities for cooperation, networking, knowledge sharing, and joint work across teams and departments. In contrast, social support included opportunities related to interpersonal relationships, social interaction, collegial assistance, and support from colleagues.

#### Collaboration

The analysis revealed that sharing work areas in ABW provided opportunities for collaboration both between and within units, fostering a strong sense of cohesion. The material also indicated that ABW created opportunities for networking and cooperation on common issues, which contributed to increased creativity, knowledge sharing, and professional development. One respondent described this as:

*"Social exchange. It becomes more cohesive and facilitates collaboration between units and within the unit."*

It was also expressed that working in close proximity to other professional groups increased the understanding of their working methods and target groups. This in turn led to synergies and collaboration. One respondent expressed this as follows: “Synergies within my area, my department, and between departments. Easier collaboration.”

#### Social support

The material showed that being gathered in the same workplace made communication between employees from different units easier and provided an environment where colleagues could promptly help and support each other. In addition, the work environment encouraged social interaction and spontaneous meetings between employees. It was expressed that discussing matters in the corridor and resolving issues “on the go” was faster and easier than communicating via chat or email. One person stated:

*“Opportunities to meet people from other departments and administrations and resolve small issues spontaneously without having to book a meeting.”*

Additionally, the possibility to establish new contacts outside the immediate team was seen as an advantage, especially for new personnel or those needing to build their social network. One participant expressed this as:

*"There is an opportunity to establish new connections outside my department […]. This can be important for newcomers or those who need to build their social network."*

### Organizational level

The third category, the organizational level, focuses on the environment and ethos within an organization (McLeroy et al., 1988). This category is built on the subcategories of *adapted facilities* and an *attractive work environment,* which are connected to this level as they reflect how the physical workspace, organizational resources, and overall workplace atmosphere influence employees' experiences and opportunities in ABW.

In the analysis, adapted facilities included workplace features that supported specific work tasks, such as work zones, meeting rooms, and technical equipment. On the other hand, an attractive work environment included broader qualities of the workplace, such as aesthetics, comfort, natural light, pleasant surroundings, and overall satisfaction with the work environment.

#### Adapted facilities

The analysis revealed that respondents considered facilities and equipment essential for performing their work tasks both individually and with other people. They highlighted the importance of having access to good technical equipment and facilities adapted for their needs, including digital solutions and screens. The proximity to IT support, internal services, and customer service in the same building was also seen as a possibility. Furthermore, it was expressed that the possibility of digitalizing the work faster, such as having a central scanning function, would make work easier and eliminate the need to carry paper around as you move about a lot in ABW.

An important organizational factor in the work environment was the provision of facilities that support environmentally friendly transportation, creating conditions to reduce environmental impact through more efficient vehicle use. This was expressed in the quote: *"Environmental benefits with a combined car and bicycle pool* *".*

Furthermore, the changing rooms were considered excellent, allowing for outdoor activities during lunch and enabling employees to cycle to and from work. It was also seen as a valuable feature that employees can park their private bicycles indoors in the garage during the winter, facilitating active transportation to and from work.

A notable advantage was also the availability of different zones (creative, middle, and quiet), as well as meeting rooms in different sizes, as it allowed employees to choose environments suited to their specific tasks and various needs.

One respondent expressed this as follows:

*"There are many advantages. Excellent workspaces, including both secluded quiet rooms and various open areas. A wide range of well-designed spaces to work in. Numerous meeting rooms of different sizes."*

While the analysis revealed that the different zones were advantageous for people who needed individual focus, collaboration, and flexibility, it was also expressed that providing additional zones for people working with sensitive information and needing more privacy would be beneficial. One person stated:

*”There are several possibilities. Division of more zones regarding confidentiality than those that exist now.”*

#### Attractive work environment

The analysis revealed that the respondents saw many opportunities in the work environment. The physical workspace was described as bright and modern, with large windows that let in plenty of natural light. It was characterized as an inspiring environment to work in, and the respondents hoped that the facilities would support both physical and mental well-being. Being in a pleasant environment was said to create a strong sense of comfort and satisfaction. One person commented: *“It’s also very nice everywhere, which creates great comfort and satisfaction, and that shouldn’t be underestimated!”.*

The close proximity to the city centre was also put forward as an advantage, as it made commuting to work and accessing the city's offerings easy. A central location was also considered beneficial for clients visiting as it may be easier to get there. One person commented: *“What is good or an opportunity is that the municipal building is centrally located so it may be easier for citizens to get here* *”.*

## Discussion

This study explored employees' perceived opportunities with ABW using the SEM as a framework. Six sub-categories were identified in the data. These were on the intrapersonal level: autonomy, health and well-being; on the interpersonal level: social support, collaboration, and on the organizational level: adapted facilities and an attractive work environment.

On the *intrapersonal level*, autonomy was highlighted as a key aspect, allowing employees to adjust their work environment according to their individual needs. This was seen as stimulating and important for maintaining focus and enhancing performance. Autonomy in the context of work reflects the extent employees can perform tasks and work in a way that aligns with their own preferences and judgement (Wan & Duffy, 2022). Previous research has also found positive effects of autonomy in general in ABW (Babapour Chafi & Rolfö, 2019). Additionally, longitudinal evidence suggests that perceived autonomy can increase over time following a transition to ABW, particularly when employees gain the flexibility to adjust their work time and location (Mache et al., 2020). Furthermore, the participants' descriptions in the present study suggest that autonomy was perceived in relation to organizational conditions, such as how the work environment was structured and how work could be organized in practice. This is in line with a review by Fatima et al. (2024), who show that organizational structures and work arrangements can influence employees' autonomy and engagement. These findings suggest that participants perceived autonomy in relation to organizational structures such as zoning, flexible work areas, and access to different workspaces.

Improved health and well-being were also seen as a possibility with ABW. The respondents perceived health benefits, such as increased physical activity during the day, consistent with previous findings (Foley et al., 2016). Other studies have highlighted challenges related to ergonomics and comfort in ABW, showing that relocation may reduce sitting comfort and optimal work posture (Wu et al., 2022). While there may be more movement in ABW while changing workstations, ergonomic adjustments can be perceived as both difficult and time-consuming (Wahlström et al., 2020). Hence, when organizations are implementing ABW, it appears to be important that their employees have good knowledge of why and how to set up their workplace (Sanaeinasab et al., 2018). These findings illustrate how employees described health and well-being in relation to organizational structures, resources, and social conditions, which is consistent with a socio-ecological perspective.

For example, Javaid et al. (2023) show that employees' well-being is not only influenced by workplace conditions, but also by relationships with colleagues and how managers lead and support their staff.

On the *interpersonal level*, the analysis revealed that sharing work areas in ABW provided opportunities for social support and collaboration between units, which led to a strong sense of cohesion. It was also indicated that ABW created opportunities for networking and cooperation on common issues. Previous research has also found that communication and collaboration increase between teams in ABW and that office design can facilitate teamwork and social exchange (Appel-Meulenbroek et al., 2011; Eismann et al., 2021). Presumably, office workers who share desks need to change workplaces repeatedly. This increases the opportunity to interact and, as a consequence, improves communication (Vos & van der Voordt, 2001). Given that most organizational tasks are now performed by teams, effective teamwork has become a critical factor in organizational success (Baker et al., 2006). Accordingly, office design should be purposefully structured to enhance collaboration between individuals (Elsbach & Bechky, 2007). However, research has also pointed out that communication within teams can be inhibited when team members are scattered in the office, and that locating colleagues may be challenging (Rolfö et al., 2018). It has been emphasized that it is important to allocate workstations properly to avoid people with jobs that have little in common sitting together (Van Der Voordt, 2004). These findings suggest that interpersonal experiences in ABW are related to both individual behaviours and organizational structures, and that social support and collaboration were important aspects of employees' experiences.

### Organizational level

On the organizational level, the physical work environment was described as bright and modern, with large windows that let in plenty of natural light. This reflects how organizational design features were initially experienced as motivating and supportive of daily work. While pleasant and well-designed environments may support comfort, satisfaction, and mood, longitudinal research has shown that favourable initial impressions of ABW do not necessarily persist over time. Previous longitudinal studies have demonstrated that although ABW environments may be perceived as attractive and inspiring, they do not always meet employees' task-related needs over time. This results in decreased use of facilities and employees adapting spaces differently than planned (Lansdale et al., 2011). From an SEM perspective, this illustrates how organizational design features initially may be related to intrapersonal experiences. However, their long-term effectiveness depends on sustained alignment between environmental conditions and work demands.

Adapted facilities, such as having access to good technical equipment, including digital solutions and screens, were also brought up as a possibility with ABW. Furthermore, the different zones that allowed people to sit in the zone most appropriate for their work task were also seen as a possibility. In this study, these organizational features were viewed as enabling conditions that helped employees adapt their work environment to different tasks. Chadburn and Smith (2015) found that IT connectivity, comfort, convenience, and good design were strong drivers of personal productivity. Furthermore, Palvalin et al. (2017) state in a study reviewing previous research in regards to the physical work environment and individual productivity, that a few of the most important factors to support employee productivity are: appropriate spatial conditions for concentration i.e., opportunities to work alone without being distracted (quiet zones), spatial conditions for communication and social interaction, fit with psychological needs such as privacy, high-quality lighting and natural daylight as well as access to advanced technology. Furthermore, studies of ABW show that more positive perceptions of the office environment are linked to better well-being and self-reported work ability, even when psychosocial factors are taken into account. This suggests that organizational design may be related to intrapersonal outcomes (Javaid et al., 2023). Taken together, these findings suggest that employees viewed organizational features in ABW as important for opportunities related to autonomy, health and well-being, collaboration, and social support.

Using SEM, this study shows how employees' perceived opportunities at intrapersonal, interpersonal, and organizational levels are closely interconnected. Experiences of autonomy, health, social support, collaboration, and access to facilities appear to be linked to both individual factors and social and organizational conditions. Overall, the study illustrates how SEM can be used as a framework for organizing and interpreting employees' perceived opportunities in ABW across multiple levels.

This interpretation is in line with previous applications of the SEM, which emphasize that individual outcomes are influenced by organizational and environmental conditions rather than individual factors alone (Habeger et al., 2022; Wu et al., 2022).

The study has both theoretical and practical significance. Theoretically, the study demonstrates the usefulness of SEM as an analytical framework for understanding employees' perceived opportunities in ABW across intrapersonal, interpersonal, and organizational levels.

Practically, the findings suggest that designing ABW with flexible spaces, supportive facilities, and attention to social and organizational factors can enhance employees' opportunities, well-being, and collaboration. For example, providing work zones suited to specific tasks, ensuring access to ergonomic and technical equipment, and creating environments that encourage team interaction and social support can improve employees' experiences. The six sub-categories identified in the study offer a useful framework for applying SEM in workplace design.

The findings also provide practical insights for managers, workplace designers, and HR professionals, as well as guidance for policymakers, on how individual, social, and organizational factors can be considered when planning and developing ABW environments. The findings should also be interpreted in light of the timing of data collection. Some of the positive perceptions may reflect novelty effects or an ongoing adjustment to the new work environment as the survey was conducted only a few months after the transition to ABW.

### Strengths and limitations

This study explores the SEM in the context of ABW and provides insights into how the SEM can be applied in flexible work environments, which, to our knowledge, has not been done before. Moreover, a strength of the study is that it has a salutogenic approach and focuses on opportunities and factors that help to identify and understand factors that promote health and a positive work experience in ABW. Further, trustworthiness was established by using Lincoln and Guba's approach of credibility, transferability, dependability, confirmability, and authenticity (Lincoln & Guba, 1985). Credibility was achieved through a systematic analysis according to Elo and Kyngäs (2008) and by using responses from the participants and iterative discussions among the authors during coding.

Transferability was strengthened by providing a clear description of the data collection process, the organizational context, and the ABW implementation, allowing readers to assess the relevance of the findings to similar settings.

Dependability and confirmability were ensured through review of written responses, reflective team discussions, documentation of decisions, and preservation of research materials to allow transparency of the research process. Finally, authenticity was strengthened by using different perspectives, including direct participant responses, and incorporating team discussions. NVivo 1.4.1 was used to systematically organize the data, ensuring coding consistency and alignment with the study objectives, and consensus was reached for all categories and subcategories.

The results reflect local conditions in northern Sweden that may not be representative of other cultural contexts, which may be considered a limitation. Nevertheless, the results may be transferable to similar Swedish public sector workplaces. Furthermore, as the participants came from one organization, it may be hard to generalize the findings to other workplaces. However, it represented different and common departments in municipal organizations. One limitation is the timing of the survey, which was conducted a couple of months after the transition. With this in mind, further follow-up studies are needed to explore whether these perceived opportunities remain stable over time or change as employees become more accustomed to the new way of working. However, this study captures real-life experiences during the early stages and is valuable from that perspective. Another limitation of this study is that, due to the salutogenic perspective, it focuses exclusively on employees' perceived opportunities in ABW. This means that potential challenges or negative experiences with ABW are not studied and could be explored in future research to provide a more balanced understanding of ABW implementation. As the analyzed survey question explicitly asked about opportunities, the material was expected to primarily generate positive responses. Therefore, the findings should not be interpreted as a complete representation of employees' experiences of ABW.

The use of a single open-ended survey question also limits the depth and contextual richness of the data compared with interview- or focus group-based qualitative studies. Responses were often brief and did not allow for follow-up questions or further exploration of participants' experiences.

Furthermore, an additional limitation is that the study did not explore how participants' demographic characteristics may influence experiences of ABW, even though participant characteristics were collected and reported.

The use of the three pre-defined SEM categories could indicate a risk of overlooking other potentially relevant themes. However, the SEM levels were used only as an analytical framework for organizing the data and did not guide data collection. Subcategories were developed inductively within each SEM level based on recurring patterns and meanings identified in the material.

## Conclusions

This study shows that the SEM levels of intrapersonal, interpersonal, and organizational factors can be used to understand employees' perceptions of opportunities in ABW. The findings highlight that autonomy and health and well-being on the intrapersonal level, collaboration and social support on the interpersonal level, as well as adapted facilities and an attractive work environment on the organizational level, are important factors shaping employees' experiences. The SEM has traditionally been used in public health research (Golden & Earp, 2012), and this study illustrates its usefulness for analyzing employees' perceived opportunities in organizational settings, especially in relation to ABW, which rarely has been done before. Workplaces wishing to adopt ABW should consider all three levels, as they are interconnected, to support employees' opportunities and well-being. The findings also provide practical insights for managers and HR professionals, as well as guidance for policymakers, on how intrapersonal, interpersonal, and organizational factors can be considered when planning and developing ABW environments.

## Data Availability

The data are not publicly available because they contain information that could compromise the privacy of research participants. Data can be requested from the corresponding author upon reasonable request.
